# Piezoelectric Ultrasonic Local Resonant Ultra-Precision Grinding for Hard–Brittle Materials

**DOI:** 10.3390/mi15101216

**Published:** 2024-09-29

**Authors:** Dawei An, Jianghui Xian, Yi Zhang, Guoqiang Cheng, Yankai Huang, Zhongwei Liang, Weiqing Huang

**Affiliations:** 1 School of Mechanical and Electrical Engineering, Guangzhou University, Guangzhou 510006, China; andavy@gzhu.edu.cn (D.A.); 2112207074@e.gzhu.edu.cn (J.X.); 2112007136@e.gzhu.edu.cn (Y.Z.); 2112207083@e.gzhu.edu.cn (G.C.); 2112207079@e.gzhu.edu.cn (Y.H.); 2 School of Computer and Information, Qiannan Normal University for Nationalities, Duyun 558000, China

**Keywords:** local resonant, ultrasonic ultra-precision grinding, material removal rate, surface quality

## Abstract

Hard–brittle materials are widely used in the optics, electronics, and aviation industries, but their high hardness and brittleness make it challenging for traditional processing methods to achieve high efficiency and superior surface quality. This study aims to investigate the application of ultrasonic local resonant grinding to sapphire to improve the efficiency and meet the requirements for the optical window in the surface roughness of the material. The resonant frequency of a piezoelectric ultrasonic vibration system and the vibration amplitude of a grinding head’s working face were simulated and tested, respectively. The results of ultrasonic grinding experiments showed that the local resonant system reduced the surface roughness parameter (Ra) of sapphire to 14 nm and improved its surface flatness to 44.2 nm, thus meeting the requirements for the ultra-precision grinding of sapphire. Compared with a conventional resonant system, the surface roughness of the sapphire ground with the local resonant system was reduced by 90.79%, its surface flatness was improved by 81.58%, and the material removal rate was increased by 31.35%. These experimental results showed that ultrasonic local resonant grinding has better effects than those of conventional ultrasonic grinding in improving surface quality and increasing the material removal rate.

## 1. Introduction

Ultrasonic machining technology is widely used in various fields as it does not generate much cutting heat in the machined material, does not cause surface burns, and achieves higher machining precision [1,2]. When ultrasonic vibration is introduced into the processing of hard–brittle materials, the processing efficiency can be effectively improved by using impacts from high-frequency vibration [3]. Sun et al. performed scratching experiments on hard–brittle materials using a Vickers indenter. Their experimental results showed that the depth of ultrasonic vibration-assisted scratching was 481 nm, and the width was 7.40 μm, while the depth of conventional scratching was 455 nm and the width was 7.06 μm. This shows that the processing efficiency for hard–brittle materials can be improved by ultrasonic vibration. At the same time, the critical depth of the transition from brittleness to toughness in the material is also increased under the action of ultrasonic vibration [4]. In order to improve the properties of Si_3_N_4_ ceramic materials, the technique of combining SiO_2_ nanofluids and an ultrasonic vibration-assisted grinding system to machine Si_3_N_4_ ceramic materials was investigated. The experimental results showed that ultrasonic vibration had the greatest influence on the tangential grinding force. Ultrasonic vibration was able to reduce the normal grinding force, the tangential grinding force, and the surface roughness by 57%, 65%, and 18%, respectively [5].

In addition, many scholars have researched grinding force modeling and material removal to reveal the mechanisms of ultrasonic vibration-assisted grinding of hard–brittle materials. Wang et al. proposed a feed-direction grinding force model for two-dimensional elliptical ultrasonic vibration-assisted grinding based on the complex motion trajectory of a single abrasive grain. Their results showed that with increasing ultrasonic amplitude, the grinding force exhibited a linearly decreasing trend, which effectively improved the surface quality of materials [6]. Chen et al. investigated the kinematics of individual abrasive grains to establish a grinding force model for ultrasonic vibration-assisted grinding. Their experimental results showed that ultrasonic vibration-assisted grinding could effectively reduce the grinding force and the surface roughness parameter (Ra) of the workpiece. It was demonstrated that the grinding force during 3D ultrasonic vibration-assisted grinding was approximately 20∼30% lower than that of 2D ultrasonic vibration-assisted grinding [7]. Zou et al. simulated the grinding of sapphire using ultrasonic vibration through the discrete element method. They experimentally verified that ultrasonic vibration could decrease the residual tensile stress and increase the residual compressive stress to inhibit the expansion of subsurface cracks. When the amplitude was increased from 4 to 8 μm, the machined surface was more homogeneous, the surface micro-pits were smaller, and the percentage of plasticity removal increased. When the amplitude increased, the inhibition of subsurface cracks was more obvious, such that high-quality sapphire materials could be processed [8]. Through computational fluid dynamics modeling and polishing experiments, Zhou et al. confirmed that abrasive particles could impact the surface of sapphire at high frequencies when driven by ultrasonic vibration. In addition, these authors proposed a novel model of material removal and proved that a pit with a depth of 0.22 nm provided evidence of two-body wear of a sapphire by studying it in situ with an atomic force microscope. The results of their experiments and simulations showed that the material removal rate increased by about 63% when the rotational speed increased from 40 to 80 r/min [9].

The importance of ultrasonic grinding is obvious when addressing the processing needs for hard–brittle materials. Sapphire is a typical hard–brittle material that is characterized by high hardness, a high melting point, high wear resistance, and stable chemical properties at high temperatures [10,11]. It is used in a variety of microelectronic devices, including light-emitting diode substrates, frequency multipliers, high-temperature sensors, and optical microscope windows [10,12,13,14,15], where the integrity of the sapphire’s surface contributes to strengthening the performance of these electronic devices [16]. The surface quality of the material determines the performance of various components [17] and, so, improving the surface quality of sapphire has become a direction of research for many scholars. It has been shown that ultrasonic grinding has unique advantages for sapphire, as it does not result in thermal damage or significant residual stresses on the workpiece, and it can significantly improve the processing efficiency and surface quality [18,19,20], which provides a new direction for the ultra-precision grinding of sapphire. However, existing ultrasonic vibration systems, which are designed using a resonant design method, are not stable. When it is affected by a load, such a system cannot output continuous and stable ultrasonic vibrations [21]. In a conventional resonant system, the resonant frequency of the entire system is usually consistent with the resonant frequency of the grinding head in a free state [22]. However, in practical applications, the grinding head cannot be in a fully free state. A piezoelectric ultrasonic vibration system designed based on the resonant method requires accurate engineering principles and fine assembly techniques. A slight deviation in the processing and assembly will change the resonant frequency of the system. These systems are susceptible to loads and self-heating, making their output performance unstable and reducing the production efficiency and surface quality [23].

To achieve a sapphire surface roughness of below 20 nm, thus ensuring that the surface quality meets the requirements for ultra-precision machining, an ultra-precisely ground sapphire can be applied to optical windows. This study proposes a method for the ultrasonic local resonant ultra-precision grinding of sapphire materials, revealing its advantages in processing hard–brittle materials. First, the structure of the piezoelectric ultrasonic vibration system was determined using an established dynamic model. Second, a simulation analysis and a performance test of the designed piezoelectric ultrasonic vibration system were carried out to determine its resonant frequency, and it was proved that the grinding head had a local resonance related to that of the amplitude transformer. Finally, the effects of different influencing factors on the material removal rate, surface roughness, and surface flatness of sapphire were explored. The ultrasonic local resonant grinding of sapphire significantly enhanced the processing efficiency and surface quality, meeting the increasing demands for sapphires in optical window applications. The optimization of this process serves as a reference for the ultra-precision grinding of other hard–brittle materials, further advancing the development of high-precision industries.

## 2. Methods and Materials

### 2.1. Design of the Ultrasonic Grinding System

**Figure 1:**
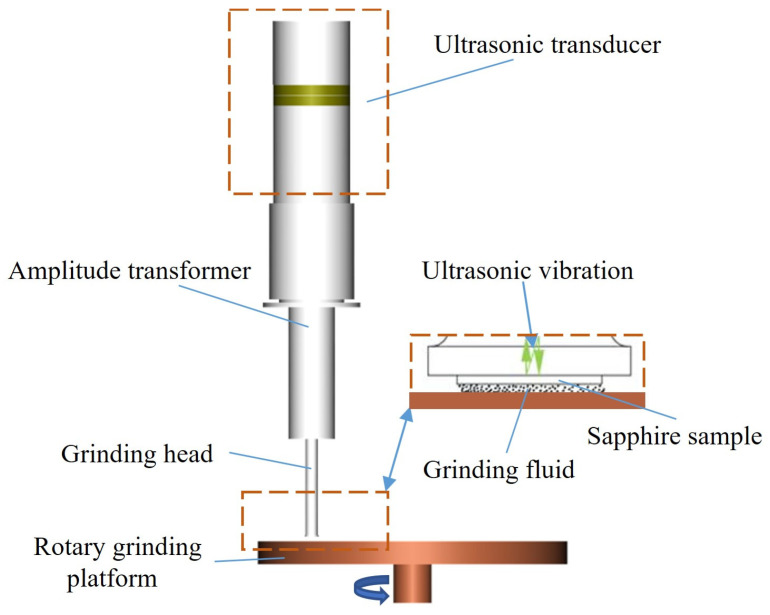
Composition of the ultrasonic grinding system.

The theory of local resonance refers to a phenomenon in which an ultrasonic grinding head can produce an individual resonance relative to the amplitude transformer. In essence, when the ratio of the cross-sectional area of the grinding head to the cross-sectional area of the amplitude transformer is sufficiently small, the resonant frequency of the entire system is nearly identical to the resonant frequency of the grinding head in the state in which one end is free and the other end is fixed, and the entire system remains in resonance [23]. The ultrasonic grinding system designed based on the local resonant theory is illustrated in Figure 1. The system was made up of several components, including an ultrasonic transducer, amplitude transformer, grinding head, and rotary grinding platform. The ultrasonic transducer was responsible for converting electrical energy into mechanical energy, while the amplitude transformer and the grinding head amplified the vibration amplitude.

**Figure 2:**
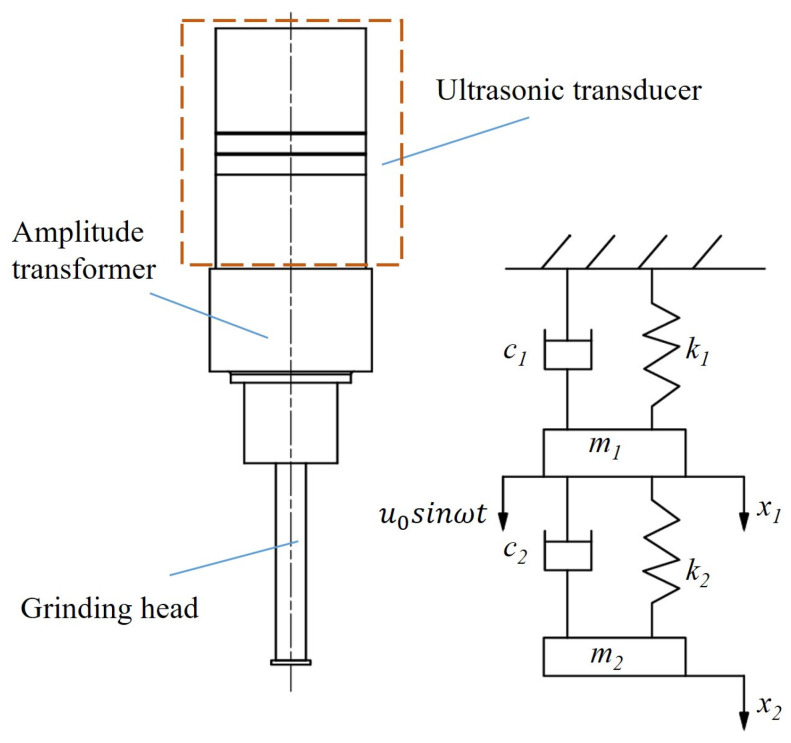
The dynamic model of the two-degree-of-freedom system.

A dynamic model composed of an amplitude transformer and grinding head was established. This model simplified the amplitude transformer and grinding head of the piezoelectric ultrasonic vibration system into an equivalent dynamic model: a mass∼spring∼damper model [24,25]. The amplitude transformer is represented by $m_{1}$∼$k_{1}$∼$c_{1}$, and the grinding head is represented by $m_{2}$∼$k_{2}$∼$c_{2}$. The dynamic model of the two-degree-of-freedom system is shown in Figure 2.

The differential equation of the system’s vibration is as follows:

(1) $$\left(\begin{array}{cc} m_{1} & 0\\ 0 & m_{2} \end{array}\right)\left(\begin{array}{c} {\ddot{x}}_{1}\\ {\ddot{x}}_{2} \end{array}\right)+\left(\begin{array}{cc} c_{1}+c_{2} & -c_{2}\\ -c_{2} & c_{2} \end{array}\right)\left(\begin{array}{c} {\dot{x}}_{1}\\ {\dot{x}}_{2} \end{array}\right)+\left(\begin{array}{cc} k_{1}+k_{2} & -k_{2}\\ -k_{2} & k_{2} \end{array}\right)\left(\begin{array}{c} x_{1}\\ x_{2} \end{array}\right)=\left(\begin{array}{c} u_{0}e^{i\omega t}\\ 0 \end{array}\right)$$

where $m_{1}$ is the quality of the amplitude transformer, $m_{2}$ is the quality of the grinding head, $k_{1}$ is the elastic coefficient of the amplitude transformer, $k_{2}$ is the elastic coefficient of the grinding head, $c_{1}$ is the damping of the amplitude transformer, $c_{2}$ is the damping of the grinding head, $x_{1}$ is the output displacement of the amplitude transformer, $x_{2}$ is the output displacement of the grinding head, and *w* is the angular frequency.

The steady-state response of the system is expressed as follows:

(2) $$\left(\begin{array}{c} x_{1}\\ x_{2} \end{array}\right)=\left(\begin{array}{c} X_{1}\\ X_{2} \end{array}\right)e^{i\omega t}$$

According to Equations (1) and (2), the vectors of the amplitude transformer and grinding head are, respectively,

(3) $$\left\{\begin{array}{c} X_{1}=\frac{-m_{2}\omega^{2}+ic_{2}\omega +k_{2}}{f\left(\omega^{2}\right)}u_{0}\\ X_{2}=\frac{ic_{2}\omega +k_{2}}{f\left(\omega^{2}\right)}u_{0} \end{array}\right.$$

where $f\left(\omega^{2}\right)=\left[-m_{1}\omega^{2}+\left(c_{1}+c_{2}\right)\omega i+k_{1}+k_{2}\right]\times \left(k_{2}-m_{2}\omega^{2}+c_{2}\omega i\right)-{\left(c_{2}\omega i-k_{2}\right)}^{2}$.

The module length of $X_{1}$ and $X_{2}$ is

(4) $$\left\{\begin{array}{c} \left|X_{1}\right|=\left|\frac{u_{0}}{f\left(\omega^{2}\right)}\right|\sqrt{{\left(-m_{2}\omega^{2}+k_{2}\right)}^{2}+{\left(c_{2}\omega \right)}^{2}}\\ \left|X_{2}\right|=\left|\frac{u_{0}}{f\left(\omega^{2}\right)}\right|\sqrt{{\left(c_{2}\omega \right)}^{2}+k_{2}^{2}} \end{array}\right.$$

The module lengths of the amplitude transformer vector and the grinding head vector, respectively, represent the magnitudes of the amplitude. The magnitude of the vibration amplitude is affected by the damping of the system [26]; when damping is not considered, there is a zero-vibration amplitude position in the system, which is at the connection between the grinding head and the amplitude transformer. When an ultrasonic transducer converts electrical energy into mechanical energy, the mechanical energy is transmitted to the amplitude transformer and the grinding head through the front cover. Upon receipt of the energy, the grinding head undergoes forced vibration, resulting in resonance when the external excitation frequency is equal to its natural frequency. The resonant frequency of a system is contingent upon the structural configuration of the system and the composition of the materials that comprise it.

The wave equation for a cylinder with a uniform cross-section is given by

(5) $$\frac{\partial^{2}\xi }{\partial x^{2}}=\frac{1}{c^{2}}\cdot \frac{\partial^{2}\xi }{\partial t^{2}}=\omega_{n}$$

where ξ(x,t) is the displacement function of a particle, *c* is the speed of sound of the grinding head, and $w_{n}$ is a frequency constant.

Equation (5) can be solved using the method of separated variables to obtain

(6) $$\xi \left(x,t\right)=\left(A_{1}\sin\frac{\omega_{n}}{c}+B_{1}\cos\frac{\omega_{n}x}{c}\right)\times \left(A_{2}\sin\omega_{n}t+B_{2}\cos\omega_{n}t\right)$$

When the grinding head is fixed at one end and free at the other,

(7) $$\left\{\begin{array}{c} \xi (0,t)=0\\ \frac{\partial \xi }{\partial x}\left(l,t\right)=0 \end{array}\right.$$

Substituting Equation (7) into Equation (6) gives

(8) $$A_{1}\frac{\omega_{n}}{c}\cos\frac{\omega_{n}l}{c}=0$$

Hence,

(9) $$\frac{\omega_{n}l}{c}=\frac{2n+1}{2}n$$

This can also be expressed as

(10) $$f_{n}=\frac{(2n+1)c}{4l},\left(n=0,1,2,\ldots \right)$$

where *n* is the serial number of the frequency, *f* is the resonant frequency, and *l* is the length of the grinding head.

In the ultrasonic vibration-assisted machining of hard–brittle materials, the resonant frequency is designed to be above 20 kHz to allow the material to be given a nanometer-scale surface roughness [5,18]. However, as the resonant frequency increases, the residual stress of the material will also increase [27]. Therefore, the resonant frequency of the piezoelectric ultrasonic vibration system was designed to be 20 kHz. Under ideal conditions, the piezoelectric ultrasonic vibration system should generate a vibration state suitable for the ultrasonic grinding of sapphire at an excitation frequency of 20 kHz. Based on the local resonant design method, the resonant frequency of the piezoelectric ultrasonic vibration system was very close to the frequency of the grinding head, which was fixed at one end and free at the other. Consequently, once the dimensions and material of the grinding head had been established, the resonant frequency of the piezoelectric ultrasonic vibration system was also determined. The grinding head was constructed from 304 stainless steel, which was selected for its exceptional corrosion resistance and superior wear resistance. The material parameters for 304 stainless steel are shown in Table 1. By substituting the parameters, the length of the grinding head was calculated to be *l* = 63 mm. Then, the diameter of the grinding head was set to *D* = 8 mm, and the diameter of the small end of the stepped amplitude transformer was set to $D_{1}$ = 30 mm. The calculated value of $D/D_{1}$, which was less than 0.3, was consistent with the local resonant design theory.

### 2.2. Finite Element Analysis and Testing

**Figure 3:**
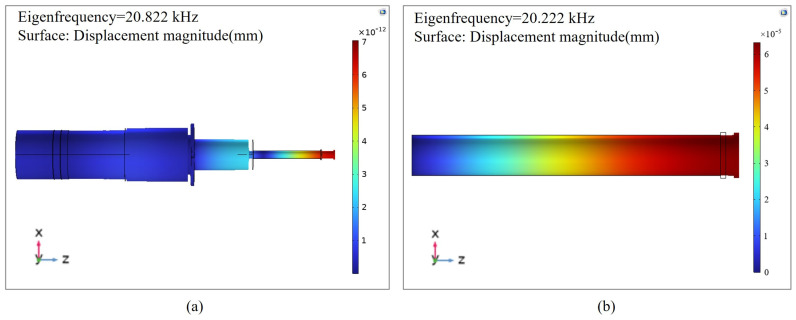
Modal analysis of the (**a**) piezoelectric ultrasonic vibration system and (**b**) grinding head.

**Figure 4:**
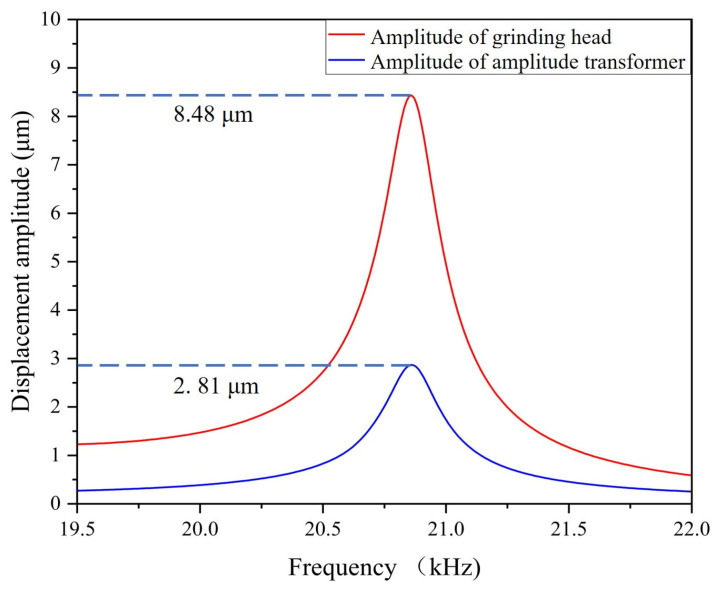
Relationship between the displacement amplitude and frequency.

The designed piezoelectric ultrasonic vibration system and grinding head were imported into the COMSOL Multiphysics 6.0 finite element analysis software. The grinding head was provided with a fixed constraint on the connecting surface of the amplitude transformer. The simulation result of the eigenfrequency of the system is shown in Figure 3a. The simulated frequency was 20.822 kHz, and the relative error in comparison with the theoretical design value was 4.11%. The error was within an acceptable range, which indicated that the design was reasonable. As shown in Figure 3b, when the grinding head was fixed at one end and free at the other end, its eigenfrequency was 20.222 kHz, which was very close to the eigenfrequency of the whole system, proving that the local resonant of the grinding head did occur. Through the finite element analysis of the eigenfrequency, we obtained the longitudinal vibration generated by the system at the natural frequency. To study the performance of the output amplitude at the natural frequency, an excitation voltage of 250 V (peak to peak) was applied between the positive and negative poles of the piezoelectric ceramic material. The frequency search range for the system was set to 19.5–22 kHz. The relationship between the displacement amplitude and frequency of the piezoelectric ultrasonic vibration system is illustrated in Figure 4.

It is demonstrated in Figure 4 that within the frequency search range of 19.5–22 kHz, the amplitude of the grinding head’s working face exhibited an initial increase, followed by a subsequent decrease, reaching a maximum of 8.48 μm at the eigenfrequency of 20.822 kHz. Concurrently, the output amplitude at the end of the amplitude transformer was 2.81 μm. The results indicated that 20.822 kHz was the resonant frequency of the system. Furthermore, the data suggested that the majority of the vibration was transmitted to the grinding head with a minor portion of the vibration being lost at the end of the amplitude transformer, resulting in the local resonant of the grinding head.

**Figure 5:**
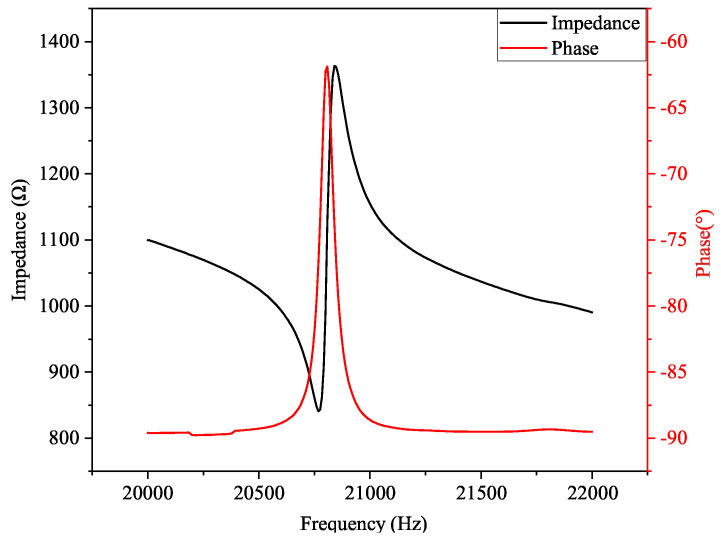
Impedance test results of the piezoelectric ultrasonic vibration system.

As the performance of the system directly affected the grinding quality, impedance analysis and amplitude performance tests were performed to verify the output performance of the system [28]. To verify the rationality of its theoretical design, the piezoelectric ultrasonic vibration system was subjected to impedance testing using a precision impedance analyzer (6630, MICROTEST, New Taipei, China). This analyzer was able to directly measure and display key parameters, such as the impedance of the device, to determine the resonant frequency of the system. The impedance test results shown in Figure 5 indicate that at a frequency of 20.768 kHz, the impedance value reached a minimum of 840.7 Ω, which represented the best frequency conductivity. Therefore, it was determined that this frequency was the resonant frequency of the piezoelectric ultrasonic vibration system at which the admittance was at its maximum, resulting in constant ultrasonic vibration.

**Figure 6:**
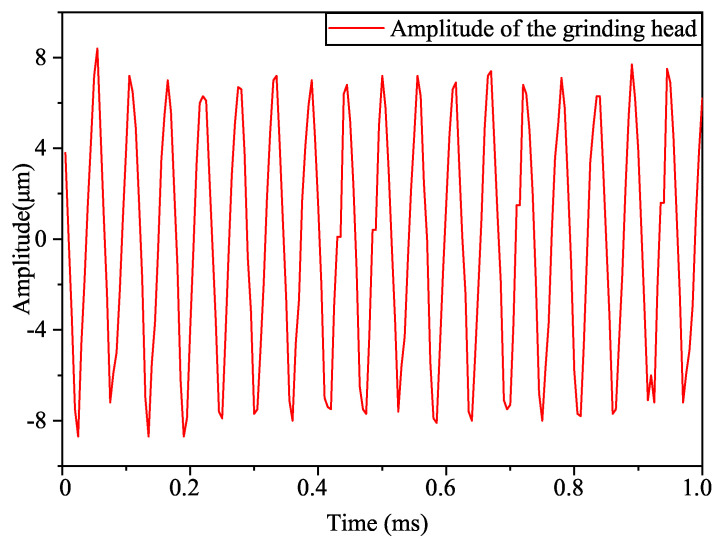
Output longitudinal vibration amplitude at 250 V.

After obtaining the resonant frequency of the system, the amplitude at this frequency had to be tested. The magnitude of the output amplitude was a crucial factor in evaluating the performance of the piezoelectric ultrasonic vibration system and was measured using a laser displacement sensor (LK-H020, KEYENCE, Osaka, Japan). The piezoelectric ultrasonic vibration system was fixed on a working table with a clamping device, and an excitation signal was connected to the positive and negative poles of the piezoelectric ceramic material so that the system generated high-frequency vibration. The laser displacement sensor was aligned to the working surface of the grinding head, and the vibration data were captured by the laser displacement sensor and displayed on a computer. The excitation frequency of the signal generator was set to 20.768 kHz, and the peak-to-peak value of the excitation voltage was set to 250 V by adjusting the power amplifier. The longitudinal amplitude curve of the grinding head’s working face within 1 ms was obtained, as shown in Figure 6. The test results showed that the output amplitude of the grinding head’s working face was 8.4 μm, and this was compared with the simulated amplitude of 8.48 μm, resulting in a relative error of only 0.952%, which indicated that the designed ultrasonic vibration device was reasonable.

### 2.3. Experimental Equipment and Methods

**Figure 7:**
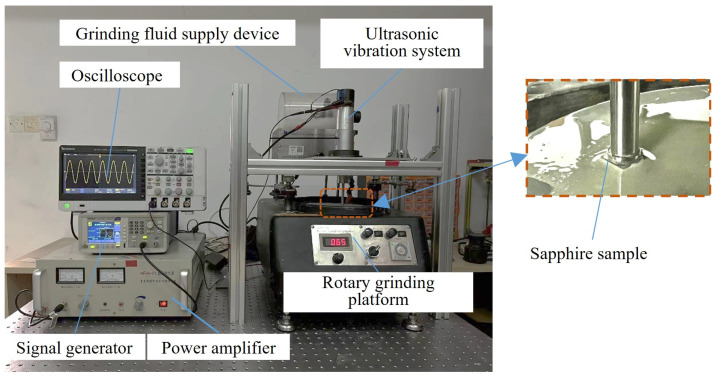
Experimental platform for ultrasonic grinding.

In order to test the effectiveness of the ultrasonic local resonant system, an ultrasonic grinding experiment was carried out. The experimental material utilized in this study was a sapphire wafer with dimensions of 10 mm × 10 mm. The initial surface flatness of the sapphire was about 518 nm, and the initial surface roughness was about 936 nm. The main parameters of the sapphire material are shown in Table 2. The main equipment required for the experiment included a grinding and polishing machine (UNIPOL-802, Shenyang Kejing, Shenyang, China), a grinding fluid supply device developed by Shenyang Kejing, a signal generator (AFG1022, Tektronix, Portland, OR, USA), a power amplifier (HFVA-61, Nanjing Foneng, Nanjing, China), and an oscilloscope (TBS2000, Tektronix, Portland, OR, USA). The excitation frequency of the signal generator was set to 20.768 kHz. The required voltage was obtained by adjusting the power amplifier. An oscilloscope was used to check whether the parameters were set correctly. Finally, the speed of the grinding platform was adjusted to the required speed, and the processing gap between the workpiece and the grinding disc was adjusted to meet the experimental requirements. The sapphire was attached to the end of the grinding head. The specific method involved the use of a heater to heat an appropriate amount of paraffin to a molten state and a brush to evenly apply the molten paraffin to the grinding head. The sapphire was placed on the grinding head coated with paraffin, and a slight pressure was applied to cause the sapphire and paraffin to become tightly bonded. The rotation of the grinding disc and the ultrasonic vibration promoted the impact of the abrasive grains on the workpiece, thus realizing the ultrasonic ultra-precision grinding of the sapphire. The experimental platform for ultrasonic grinding is shown in Figure 7. Given the potential influence of factors such as turbulent liquid motion, abrasive impact, and ultrasonic cavitation on material removal [29,30,31], the experiment was designed to investigate the effects of variations in the processing gap, rotational speed, and abrasive particle concentration (SiC) on the material removal rate and surface roughness of the sapphire. The material removal rate (MRR) was calculated by measuring the difference in mass before and after grinding the sapphire [32]. The formula for calculating the MRR is as follows:

(11) $$MRR=\frac{\Delta m\cdot 10^{7}}{\rho \cdot s\cdot t}$$

where Δ*m* is the difference in mass before and after grinding the sapphire, ρ is the density of the sapphire, and s is the surface area of the sapphire.

The surface roughness, two-dimensional surface morphology, and three-dimensional surface morphology of the sapphire before and after the experiment were tested using a surface profiler (XM-200, LUOYANG BEARING RESEARCHINSTITUTE, Luoyang, China), an optical super-depth-of-field microscope (DVM6, Leica Microsystems, Wetzlar, Germany), and an atomic force microscope (Dimension Icon, BRUKER, Billerica, MA, USA), respectively. A conventional resonant system was included as a control to make the experiment more convincing. The conventional resonant system was designed using the resonant design method, and it had a resonant frequency of 19.76 kHz. In order to ensure that the output amplitudes of the two systems were consistent, the excitation voltage of the conventional resonant system was adjusted to 500 V (peak to peak). At this time, the output amplitude was 8.42 μm.

## 3. Results

### 3.1. Surface Roughness and Material Removal Rate

**Figure 8:**
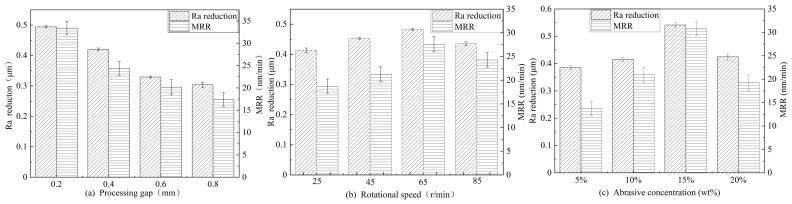
Therelationship between the surface roughness and material removal rate of sapphire and various factors: (**a**) processing gap, (**b**) rotational speed, and (**c**) abrasive particle concentration.

In the ultrasonic grinding experiment using the local resonant system, four parameters were established for each influencing factor to investigate the material removal rate and surface roughness of the sapphire by controlling individual variables. The results of the experimental investigation of each influencing factor are presented in Figure 8. It can be observed that the highest values of the reduction in the surface roughness parameter (Ra) and the highest material removal rate were achieved when the processing gap was 0.2 mm (the distance between the grinding surface of the sapphire samples and the rotary grinding platform was 0.2 mm), the speed of the rotary grinding platform was 65 r/min, and the concentration of the abrasive particles was 15 wt%.

**Figure 9:**
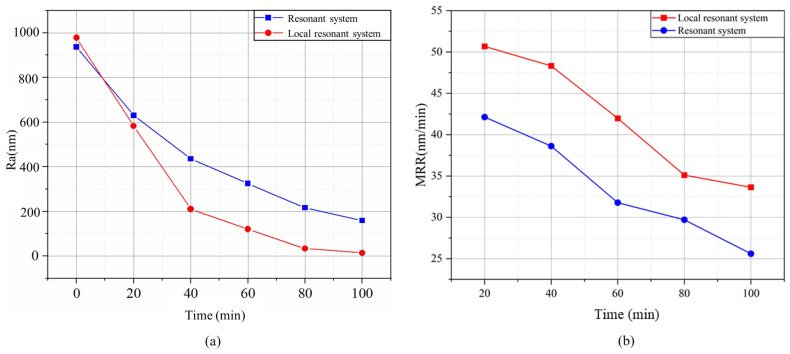
Changes with processing time: (**a**) Ra; (**b**) MRR.

Based on the above experimental results, the optimal processing parameters were obtained. In order to explore whether the designed piezoelectric ultrasonic local resonant system had more advantages in improving the material removal rate and reducing the surface roughness, a comparitive ultrasonic grinding experiment was designed based on the parameters shown in Table 3. The ultrasonic grinding of the sapphire was performed with a processing time of 100 min, and the surface roughness was measured every 20 min with a profilometer. The experimental results are shown in Figure 9.

Equation (11) was used to calculate the material removal rate for both groups of sapphires every 20 min. As shown in Figure 9, both the resonant and the local resonant systems were able to reduce the surface roughness of the sapphire during ultrasonic grinding. In the experiment using the resonant system, the surface roughness parameter (Ra) of the sapphire decreased from 936.1 to 152 nm after 100 min, the roughness decreased by 784.1 nm, and the material removal rate was 25.61 nm/min. In the experiment using the local resonant system, the surface roughness parameter (Ra) of the sapphire decreased from 977.5 to 14 nm, the roughness decreased by 963.5 nm, and the material removal rate was 33.64 nm/min within the same experimental time. The experimental results suggest that the local resonant system outperformed the resonant system on the sapphire in terms of better surface roughness and material removal rate.

### 3.2. Surface Morphology

**Figure 10:**
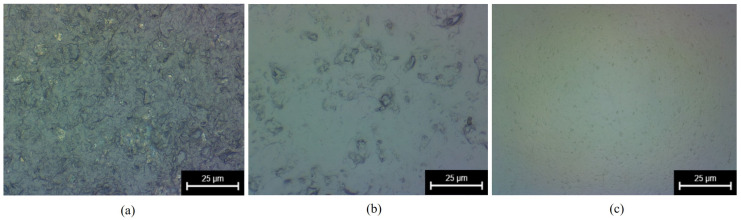
The 2D surface morphology: (**a**) original, (**b**) after grinding with a conventional resonant system, and (**c**) after grinding with the local resonant system.

**Figure 11:**
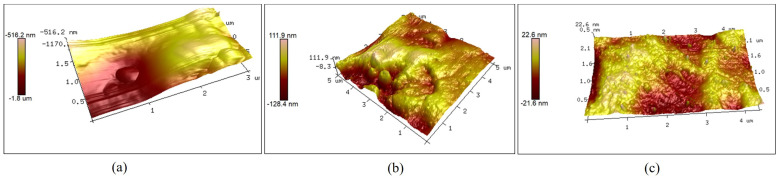
The 3D surface morphology: (**a**) original, (**b**) after grinding with a conventional resonant system, and (**c**) after grinding with the local resonant system.

As the surface microstructure is a key factor in evaluating the quality of a material, an optical super-depth-of-field microscope was used to observe the original two-dimensional surface morphology of the sapphire and the two-dimensional surface morphology after experiments with two different systems. The observed results are shown in Figure 10. In addition to the two-dimensional surface morphology of the sapphire, it was also necessary to test its three-dimensional surface morphology to compare the surface flatness after grinding using the two different systems. The three-dimensional surface morphology of the sapphire was observed using atomic force microscopy, as shown in Figure 11.

As shown in Figure 10, relative to the original sapphire wafer, both systems improved the two-dimensional surface morphology of the sapphire with the effect of ultrasonic vibration as the processing time increased. However, the sapphire ground with the local resonant system achieved a mirror effect, as shown in Figure 10c, and it had significantly improved surface smoothness. As shown in Figure 11b, the three-dimensional surface morphology of the sapphire was significantly enhanced through the use of a resonant system. Nevertheless, noticeable pits were observed with a height difference of approximately 240 nm. Meanwhile, the sapphire surface ground with the local resonant system was smoother and had a height difference of about 44.2 nm. It can be seen that compared with the use of the resonant system for grinding, the use of the local resonant system grinding improved the surface flatness of the sapphire by 81.58%. Further, the large-area pits on the surface of the sapphire basically disappeared, and the surface quality was significantly higher than that of the sapphire ground with the resonant system. The sapphire ground with the local resonant system exhibited obvious advantages in surface roughness, material removal rate, and surface morphology compared with the sapphire ground with the resonant system.

## 4. Discussion

It can be observed, from Figure 8, that reductions in the surface roughness parameter (Ra) and the highest material removal rate were achieved when the processing gap was 0.2 mm, the speed of the rotary grinding platform was 65 r/min, and the concentration of the abrasive particles was 15 wt%. This was because as the processing gap increased, the kinetic energy of the abrasive particles decreased, and the impact of the abrasive particles in the grinding fluid on the sapphire decreased. When the rotation speed of the grinding platform and the concentration of abrasive particles increased within a certain range, the number of abrasive particles scraping the surface of the workpiece per unit of time increased, which is conducive to improving the material removal rate and reducing the surface roughness. When the rotation speed was 85 r/min and the abrasive concentration was 20 wt%, the material removal rate decreased. This was because when the rotation speed of the grinding platform and the concentration of the abrasive particles exceeded a certain range, the interaction between the abrasive particles was obvious, resulting in a decrease in the number of effective cuttings. An excessive abrasive particle concentration would lead to the crowding of abrasive particles, an increase in friction, and heat accumulation between abrasive particles, which would reduce the grinding efficiency and may lead to an increase in the surface roughness.

Another study showed that excellent performance can be obtained when the surface roughness of the optical element is less than 48 nm [33]. Additionally, when the surface roughness of the material is below 20 nm and the surface flatness is less than 100 nm, the grinding surface is uniform, and the material meets the requirements for ultra-precision machining [34]. The piezoelectric ultrasonic vibration system designed using the local resonant theory reduced the surface roughness parameter (Ra) of the sapphire surface to 14 nm, and the surface flatness was improved to 44.2 nm, thus meeting the requirements for the ultra-precision grinding of sapphire. The improvement of the sapphire surface quality after ultrasonic grinding also met the material requirements for optical windows. In existing ultrasonic vibration-assisted machining systems, the surface roughness can be reduced to 44∼450 nm [5,35,36]. The surface roughness parameter (Ra) of the sapphire ground with the resonant system used in this study was reduced to 152 nm. The experimental results of the sapphire ground with the resonant system were consistent with the results obtained with the existing technology, and a surface roughness parameter (Ra) of 14 nm was obtained for the sapphire ground with the local resonant system. The surface flatness of a sapphire that has undergone non-contact ultrasonic grinding is about 1.6 μm [3]. As shown in Figure 11c, ultrasonic local resonant grinding was able to improve the surface flatness to 44.2 nm. The experimental results showed that ultrasonic local resonant grinding was more conducive to improving the surface quality of sapphire. In addition, the local resonant system avoided the problems of processing damage. The main reason was that the piezoelectric ultrasonic vibration system designed with the resonant method resulted in an unstable output amplitude and an uneven impact of abrasive particles on the workpiece’s surface. This led to uneven material removal during ultrasonic grinding and other negative outcomes, such as noticeable pits. However, the local resonant system effectively addressed this issue by providing a more stable output amplitude, thus improving the surface quality of the sapphire.

## 5. Conclusions

After ultrasonic local resonant grinding, the sapphire met the requirements for optical windows in terms of surface quality, which reached an ultra-precise level. The conclusions drawn from this study are as follows:

(1) The system’s local resonant occurred at the grinding head with a resonant frequency of 20.768 kHz. Under 250 V of peak-to-peak excitation, the longitudinal vibration amplitude was 8.4 μm.
(2) The optimal process parameters for the ultrasonic local resonant grinding of a sapphire were a processing gap of 0.2 mm, a grinding disc speed of 65 r/min, and an abrasive particle concentration of 15 wt%.
(3) The local resonant system reduced the surface roughness parameter (Ra) of sapphire to 14 nm and improved the surface flatness to 44.2 nm, which showed that the designed system met the requirements for the ultrasonic ultra-precision grinding of sapphire.
(4) Compared with a conventional resonant system, the surface roughness of the sapphire ground with the local resonant system was reduced by 90.79%, the surface flatness was improved by 81.58%, and the material removal rate was increased by 31.35%.

The piezoelectric ultrasonic local resonant system designed in this study improves the surface quality of sapphire and serves as a reference for the ultra-precision machining of other hard–brittle materials. This technology is expected to advance ultra-precision machining to meet higher process demands in the semiconductor industry.

## Figures and Tables

**Table 1:** Parameters for 304 stainless steel.

Density	Velocity of Sound	Elastic Modulus	Poisson’s Ratio
7930 (kg/m^3^)	4941 (m/s)	200 (GPa)	0.3

**Table 2:** The parameters of the sapphire materials.

Density	Mohs Hardness	Poisson’s Ratio	Melting Point	Young’s Modulus
3.98 (g/cm³)	9	0.27–0.29	2040–2050 (°C)	345 (GPa)

**Table 3:** Parameters of the ultrasonic grinding experiment.

Frequency	Voltage	Processing Gap	Rotational Speed	Abrasive Particle Concentration
20.768 (kHz)	250 $\left(V_{pp}\right)$	0.2 (mm)	65 (r/min)	15 (wt%)

## Data Availability

Data are contained within the article.
